# Using Primary Care Clinical Text Data and Natural Language Processing to Identify Indicators of COVID-19 in Toronto, Canada

**DOI:** 10.1371/journal.pdig.0000150

**Published:** 2022-12-07

**Authors:** Christopher Meaney, Rahim Moineddin, Sumeet Kalia, Babak Aliarzadeh, Michelle Greiver

**Affiliations:** 1 Department of Family and Community Medicine, Faculty of Medicine, University of Toronto, Toronto, Canada; 2 North York Family Health Team, North York General Hospital, Toronto, Canada; McGill University, CANADA

## Abstract

The objective of this study was to investigate whether a rule-based natural language processing (NLP) system, applied to primary care clinical text data, could be used to monitor COVID-19 viral activity in Toronto, Canada. We employed a retrospective cohort design. We included primary care patients with a clinical encounter between January 1, 2020 and December 31, 2020 at one of 44 participating clinical sites. During the study timeframe, Toronto first experienced a COVID-19 outbreak between March-2020 and June-2020; followed by a second viral resurgence from October-2020 through December-2020. We used an expert derived dictionary, pattern matching tools and contextual analyzer to classify primary care documents as 1) COVID-19 positive, 2) COVID-19 negative, or 3) unknown COVID-19 status. We applied the COVID-19 biosurveillance system across three primary care electronic medical record text streams: 1) lab text, 2) health condition diagnosis text and 3) clinical notes. We enumerated COVID-19 entities in the clinical text and estimated the proportion of patients with a positive COVID-19 record. We constructed a primary care COVID-19 NLP-derived time series and investigated its correlation with independent/external public health series: 1) lab confirmed COVID-19 cases, 2) COVID-19 hospitalizations, 3) COVID-19 ICU admissions, and 4) COVID-19 intubations. A total of 196,440 unique patients were observed over the study timeframe, of which 4,580 (2.3%) had at least one positive COVID-19 document in their primary care electronic medical record. Our NLP-derived COVID-19 time series describing the temporal dynamics of COVID-19 positivity status over the study timeframe demonstrated a pattern/trend which strongly mirrored that of other external public health series under investigation. We conclude that primary care text data passively collected from electronic medical record systems represent a high quality, low-cost source of information for monitoring/surveilling COVID-19 impacts on community health.

## Author summary

The coronavirus disease 2019 (COVID-19) pandemic was caused by the viral respiratory pathogen severe acute respiratory syndrome coronavirus-2 (SARS-CoV2). In many countries COVID-19 remains a persistent threat to human health/well-being, with the capability of inducing continued human, social, and economic loss. In this research study we sought to investigate whether high quality clinical narrative data, passively captured (in a cost-effective manner) from primary care patient electronic medical records, could be used as an indicator of COVID-19 viral activity. We applied a rule-based natural language processing (NLP) system to infer the COVID-19 positivity status of primary care text records. Using the rule-based NLP system we identified hundreds of unique COVID-19 entities, and thousands of COVID-19 positive documents, across millions of clinical text records. Over the study timeframe (January 1, 2020 through December 31, 2020), 2.3% of primary care patients had a COVID-19 positive document in their electronic medical record. We observed variation in COVID-19 positivity status by age, gender, and clinic location. The rule-based NLP system performed well on internal validation; further, our NLP derived COVID-19 positivity series demonstrated patterns/trends which strongly mirrored that of other external public health series under investigation. We conclude that primary care text data passively collected from electronic medical record systems represent a high quality, low-cost source of information for monitoring/surveilling COVID-19 impacts on community health.

## Introduction

The coronavirus disease 2019 (COVID-19) pandemic is caused by the viral respiratory pathogen severe acute respiratory syndrome coronavirus-2 (SARS-CoV2). Globally it is estimated that over 600 million SARS-CoV2 infections have occurred and that over 6.5 million people have died because of COVID-19 [1]. In many countries COVID-19 remains a persistent threat to human health/well-being, with the capability of inducing continued human, social, and economic loss.

Currently, a confluence of viral, socio-behavioural, and economic factors create a landscape which potentially exacerbates viral transmission and places populations at continued risk of COVID-19 infection. Outside of human control, there exists considerable uncertainty regarding the natural evolution of the SARS-CoV2 virus, and novel variants of concern continue to emerge [2,3,4]. When dealing with novel COVID-19 variants of concern, the science regarding transmission characteristics, protection conferred from available vaccines, and clinical presentation/severity resulting from infection are necessarily incomplete/evolving. From a sociological perspective, vaccine hesitancy, coupled with misinformation regarding risks/benefits of vaccines threaten efforts to achieve herd immunity against COVID-19 [5,6,7]. Further, global inequities make it that many nations lack the economic resources to procure and distribute vaccines for their populations [8]. As the pandemic extends, there is a risk that continued public adherence with non-pharmaceutical interventions aimed at mitigating viral transmission will wane (e.g. mask wearing, social distancing, and lockdowns) [9,10]. As a result of these aforementioned factors, and a multitude of others, in many regions across the globe COVID-19 may remain a continued threat to community health.

Working from a hypothesis that we will continue to live with the threat of COVID-19 for the foreseeable future, it becomes paramount that governments and public health authorities develop technologies to rapidly identify and respond to viral re-emergence in their local communities. Tools should assist decision makers determine when/how to re-implement non-pharmaceutical interventions, when to increase procurement/distribution of vaccine inventories, and when/how to expand hospital/ED/ICU capacity. Throughout the pandemic public health researchers have sought novel scientific technologies/data-sources which may act as leading indicators of viral activity, including: cell phone mobility data [11], search history and social media posts [12], data from contact tracing applications [13], environmental data sources (e.g. COVID-19 RNA from wastewater sources) [14], and health system electronic medical record data [15,16].

In this study we seek to investigate whether high quality clinical narrative data, passively captured (in a cost-effective manner) from primary care patient electronic medical records, can be used as an indicator of COVID-19 viral activity. We apply a rule-based COVID-19 natural language processing (NLP) system to infer the COVID-19 positivity status of primary care text records. Preliminary study objectives relate to the identification of COVID-19 entities in clinical text streams, assessment of COVID-19 positivity status at a document/patient level, and exploration of the agreement in COVID-19 positivity status across mined primary care text streams. Further, we review and manually label a random sample of primary care clinical text documents, and conduct an internal validation study, estimating the operating characteristics of the COVID-19 biosurveillance system employed in our study setting (i.e. estimating sensitivity, specificity, positive predictive value and negative predictive value). The main objective of the study involves the construction of a primary care NLP-derived COVID-19 indicator series, and assessment of its correlation with other available COVID-19 series (obtained from Toronto Public Health), including: 1) lab confirmed COVID-19 cases, 2) COVID-19 hospitalizations, 3) COVID-19 ICU admissions, and 4) COVID-19 intubations.

## Methods

### Study setting and context

The study setting is Toronto, Canada. Toronto is the fourth largest metropolitan city in North America and is one the most multicultural and socio-economically diverse cities of in the world. Since the WHO declared COVID-19 a pandemic of global concern in March 2020, Toronto has undergone multiple distinct waves of COVID-19 infection and at times the city has been considered a regional COVID-19 hotspot [17]. As of writing (September 2022) Toronto has recorded >360k lab confirmed COVID-19 infections and recorded >4.4k deaths. Provincial/municipal governments have attempted to mitigate pandemic related threats via mass vaccination campaigns, a combination of non-pharmaceutical interventions, including: promotion of basic infection control measures (e.g. hand washing, use of face masks and other personal protective equipment), contact tracing, social distancing and when necessary strict lockdown measures (i.e. school/business closures, restrictions on large-scale gatherings, and inter-jurisdictional travel advisories). Beginning in 2021, Toronto initiated a mass vaccination campaign, and as of writing (September 2022) Toronto has one of the highest vaccination uptake rates in the world (92.4% of residents aged 12+ with 1 vaccine dose, 89.8% residents aged 12+ with 2 vaccine doses, and 59.2% of residents aged 12+ with 3 vaccine doses) [17]. Currently, there exists considerable uncertainty regarding novel COVID-19 variants of concern, long-term vaccine effectiveness (including the need for continued booster shots and/or herd immunity), and the possibility of additional waves of COVID-19 infection in 2022 (and beyond).

### Study design, data sources, and inclusion/exclusion criteria

The study employs a retrospective open cohort design. The study start date is January 1, 2020 and the study end date is December 31, 2020. Data are collected from primary care physicians (and their associated patients) contributing data to the University of Toronto Practice Based Research Network (UTOPIAN: https://www.dfcm.utoronto.ca/landing-page/utopian). Data are collected at a record/encounter level, with (potentially multiple) encounters hierarchically nested within patients, physicians, and clinics.

Several ID variables are used in the study, including encounter-level IDs (bill ID, lab ID, health condition diagnosis ID, and clinical note ID), patient ID, physician ID and site/clinic ID. Three main text data streams are utilized in the study: 1) lab text, 2) health condition diagnosis text, and 3) clinical notes. Lab text describes laboratory test orders/results. Health condition diagnosis text contain short narrative descriptions of physician verified health states. Clinical notes are rich/descriptive variable length text documents describing aspects of the patient-physician clinical encounter. Each of the text data streams are measured on an encounter level. Patient age and sex are two demographic variables used for characterizing the cohort under study.

We include all records (and associated patients) who experience at least one clinical encounter with the primary healthcare system over our study timeframe. We define a valid encounter as an observed billing ID, lab ID, health condition diagnosis ID, or clinical note ID occurring over the study period which generates a non-null data value. Using site/clinic level postal code information we include only sites from Toronto, Canada (leading character M in the postal code). We exclude patients if they are missing age, sex, or postal code.

### A natural language processing system for COVID-19 document classification and biosurveillance

We utilize a rule based natural language processing (NLP) system to identify (at an encounter/document level) primary care text streams containing utterances pertaining to COVID-19 [15]. The NLP system uses pattern matching and expert curated dictionaries to identify COVID-19 related entities in clinical text streams. In addition, the system uses algorithms such as the ConText algorithm [18] to disambiguate the linguistic circumstances under which the COVID-19 entity occurred (e.g. who experienced the COVID-19 related entity, whether the COVID-19 related entity occurred or was negated, whether the utterance was not related to a patient COVID-19 diagnosis, and generally, whether there existed uncertainty regarding the COVID-19 entities occurrence). At a document level, each primary care clinical record is classified as: 1) COVID-19 positive, 2) COVID-19 negative, or 3) unknown COVID-19 status. The dictionaries, contextual analyzer, and document classification logic used in Chapman et al [15] were not modified prior to utilization in our study context. We apply the COVID-19 biosurveillance tool to three clinical text streams from the UTOPIAN primary care electronic medical record: 1) lab text, 2) health condition diagnosis text, and 3) clinical notes. The tool was originally developed and validated at the United States Veteran’s Affairs Hospital system [15]. The operating characteristics of the NLP system were exceptional, with internal validation reporting 94.2% sensitivity and 82.4% positive predictive value. Computational implementation of the Python based COVID-19 biosurveillance tool is further described in the original authors GitHub repository [https://github.com/abchapman93/VA_COVID-19_NLP_BSV].

### Statistical methods

We identify COVID-19 entities in each primary care clinical text stream using an unmodified version of the COVID-19 biosurveillance system developed by Chapman et al [15]. At a record level we classify each free text clinical document as 1) COVID-19 positive, 2) COVID-19 negative, or 3) unknown COVID-19 status. We report descriptive characteristics regarding the number/proportion of positive COVID-19 documents at an encounter-level. We aggregate encounter level COVID-19 document classifications to a patient level and describe the number of patients whose primary care clinical notes contain at least one positive COVID-19 utterance. At a patient-level, we cross-tabulate derived COVID-19 positive indicator vectors measured from each of the text streams under study and describe agreement in COVID-19 ascertainment across data sources. Finally, we investigate (at a patient-level) whether demographic variables such as age, sex, or clinic location are associated with COVID-19 positivity status.

We conduct an internal validation study to estimate the operating characteristics of the COVID-19 biosurveillance system in our study setting. A single researcher/biostatistician (CM) reviewed 6000 primary care documents: 1) 2000 lab texts, 2) 2000 health condition diagnosis texts and 3) 2000 clinical notes. We aggregate the human labelled notes across each of the three primary care text streams and estimate sensitivity, specificity, positive predictive value and negative predictive value of the biosurveillance system. We construct exact 95% binomial confidence intervals about each of the aforementioned statistics.

Using descriptive time series methods, we investigate temporal variation in the number of COVID-19 positive documents in the electronic medical record over our study timeframe. We discretize time in 53 weekly bins, from January 1, 2020 through December 31, 2020. We create an overall count of the number of COVID-19 positive utterances/occurrences by summing the number of positive document classifications from each of the three text streams (1. lab text, 2. health condition diagnosis text, and 3. clinical notes). Using descriptive time series plots we visually compare how our NLP derived primary care COVID-19 positivity series correlates with other known COVID-19 time series from Toronto Public Health, including: 1) lab confirmed COVID-19 cases, 2) COVID-19 hospitalizations, 3) COVID-19 ICU admissions, and 4) COVID-19 intubations [17].

### Ethics

This study received ethics approval from North York General Hospital Research Ethics Board (REB ID: NYGH (20–0014).

## Results

### Descriptive characteristics of study sample

During the study timeframe 196,440 patients had contact—in-person, email, phone or video—with their primary care provider. The majority of patients observed in the sample are female (58.8%). In terms of age structure: 14.1% of patients are 0–18 years old, 27.1% of patients are 18–40 years old, 37.2% of patients are 40–65 years old, 18.4% of patients are 65–85 years old and 3.2% of patients are >85 years old. Table 1 describes demographic characteristics associated with lab text, health condition diagnosis text and clinical notes.

**Table 1:** Descriptive characteristics of lab texts, health condition diagnosis texts and clinical notes included in the study sample, measured on a record/encounter-level.

	Lab Text	Health Condition Diagnosis Text	Clinical Notes
Number records	2,360,957	107,075	454,503
Age - 0–18 years - 18–40 years - 40–65 years - 65–85 years - >85 years	40,522 (1.7%) 420,851 (17.8%) 997,457 (42.3%) 770,285 (32.6%) 131,842 (5.6%)	7,800 (7.3%) 25,887 (24.2%) 41,110 (38.4%) 27,398 (25.6%) 4,880 (4.5%)	34,324 (7.6%) 101,081 (22.2%) 170,701 (37.6%) 120,888 (26.6%) 27,509 (6.0%)
Sex - Male - Female	948,508 (40.2%) 1,412,449 (59.8%)	40,040 (37.4%) 67,035 (62.6%)	164,780 (36.2%) 289,723 (63.8%)

### COVID-19 clinical entities and document classifications in primary care clinical text streams

We identified a diverse variety of COVID-19 entities in the three primary care electronic medical record text streams investigated in this study. Both the list of specific entities/tokens and the most-frequently occurring entities/tokens identified vary according to the text stream under consideration. Using document classification logic, the COVID-19 biosurveillance system classifies documents as 1) COVID-19 positive, 2) COVID-19 negative, or 3) unknown COVID-19 status. We report the percentage of documents in each COVID-19 category in Table 2.

**Table 2:** COVID-19 document classifications and COVID-19 entities/tokens identified from each primary care clinical text stream: lab text, health condition diagnosis text and clinical notes.

	Lab Text (N = 2,360,957 records)	Health Condition Diagnosis Text (N = 107,075 records)	Clinical Notes (N = 454,503 records)
COVID-19 Document Classification - COVID-19 Positive - COVID-19 Negative - Unknown COVID-19 Status	1,976 (0.1%) 2,296,462 (97.3%) 62,519 (2.6%)	539 (0.5%) 105,380 (98.4%) 1,156 (1.1%)	4,018 (0.9%) 411,093 (90.4%) 39,392 (8.7%)
Number of Unique COVID-19 Entities	277	121	644
Top-10 Most Frequent COVID-19 Entities - Rank 1 - Rank 2 - Rank 3 - Rank 4 - Rank 5 - Rank 6 - Rank 7 - Rank 8 - Rank 9 - Rank 10	COVID-19 COVID19 Novel Coronavirus CoronaVirusN2019 COVID Novel coronavirus SARS-CoV-2 2019-nCoV coronavirus 2019-nCov	COVID-19 COVID COVID-19 Confirmed covid COVID19 Covid-19 Covid COVID positive COVID+ Covid +ve	COVID covid COVID-19 Covid Covid-19 COVID19 covid-19 coronavirus COVID-19 coronavirus covid19

### Patient level COVID-19 status, agreement of COVID-19 status by source, and correlates of COVID-19

196,440 unique patients had at least one clinical event recorded in their primary care electronic medical record over our study timeframe. A total of 1,573 (0.8%) of these patients had a COVID-19 positive lab text in their medical record, 516 (0.3%) of these patients had a COVID-19 positive health condition diagnosis text in their medical record, and 3,022 (1.5%) had a COVID-19 positive clinical note in their medical record. We constructed a binary composite COVID-19 status variable (at a patient-level), based on whether any of the text streams under consideration indicate COVID-19 positive status. A total of 4,580 (2.3%) of patients with at least one primary care contact/record experienced at least one COVID-19 positive document in their electronic medical record.

In this study, we observe increasing COVID-19 positivity status with increasing age. We observe a positive indication of COVID-19 in 1.3% of patient 0–18 years old, 2.4% of patients 18–40 years old, 2.4% of patients 40–65 year old, 2.6% of patients 65–85 years old and 3.3% of patients 85 years or older. In our sample, we observe that females were more likely than males to record a COVID-19 positive diagnosis over our study timeframe (2.5% in females vs. 2.1% in males). COVID-19 positivity status varied across geographic regions of Toronto. In one clinic 8.9% of patients observed over the study timeframe had a COVID-19 positive indicator in their electronic medical record; whereas, in the least impacted clinic only 0.09% of patients presenting over the pandemic year had a recorded COVID-19 positive indicator in the electronic medical record.

At a patient level we descriptively evaluate agreement between COVID-19 positive status as estimated across each of the three different electronic medical record text streams: 1) lab text, 2) health condition diagnosis text and 3) clinical notes. Table 3 highlights low/limited agreement in COVID-19 positivity status as identified from each of the three text streams.

**Table 3:** A three-way cross-tabulation comparing the number of COVID-19 positive indications (at a patient-level) from the lab text, health condition diagnosis text and clinical note data streams.

	Lab Text: COVID-19 Negative		Lab Text: COVID-19 Positive	
	Health Condition: COVID-19 Negative	Health Condition: COVID-19 Positive	Health Condition: COVID-19 Negative	Health Condition: COVID-19 Positive
Clinical Notes: COVID-19 Negative	191,860	288	1,136	134
Clinical Notes: COVID-19 Positive	2,659	60	269	34

### Internal validation of a COVID-19 biosurveillance system in the context of UTOPIAN

We conducted an internal validation study to investigate operating characteristics of the COVID-19 biosurveillance system developed by Chapman et al [15] in the context of our study setting—primary care clinical practices in Toronto, Canada. Using data from the United States Veteran’s Affairs health system, Chapman et al [15] estimate the sensitivity of their COVID-19 biosurveillance algorithm to be 94.2% and the positive predictive value to be 82.4%.

For each of the three text streams under consideration in our study (1. lab text, 2. health condition diagnosis text, and 3. clinical notes) we randomly sampled N=2000 documents for inclusion in our internal validation study. A single researcher/biostatistician (CM) reviewed each of the randomly sampled free text notes and labelled them as COVID-19 positive versus COVID-19 negative. For each of the three text streams, we compared the human labelled COVID-19 classifications versus the algorithm derived COVID-19 classifications. We report the findings from our internal validation study in Table 4 and Table 5.

**Table 4:** Internal validation of the COVID-19 biosurveillance system (comparing human labelled document classifications versus algorithm derived document classifications) when applied to the following primary care text streams: lab text, health condition diagnosis text, and clinical notes.

Text Stream	Human Label	Algorithm Prediction COVID-19 Negative	Algorithm Prediction COVID-19 Unknown	Algorithm Prediction COVID-19 Positive
Lab Text	Negative	1930	68	0
	Positive	0	0	2
Health Conditions Diagnosis Text	Negative	1975	4	0
	Positive	0	9	12
Clinical Notes	Negative	1753	235	0
	Positive	0	6	6

**Table 5:** Internal validation of the COVID-19 biosurveillance system relative to the aggregated UTOPIAN lab text, health condition diagnosis text and clinical note data sources (N = 6000 independent free text documents).

	Algorithm Prediction COVID-19 Negative	Algorithm Prediction COVID-19 Unknown	Algorithm Prediction COVID-19 Positive
Human Label: Negative	5658	307	0
Human Label: Positive	0	15	20

For the lab text stream (Table 4), only two positive COVID-19 notes were identified on human review, both of which were also identified as positive by the COVID-19 biosurveillance algorithm. In the lab text, 68 notes were identified as unknown COVID-19 status by the biosurveillance algorithm, all of which were identified as negative on human review. Several of these 68 notes represented truly negative lab reports, where COVID-19 lab tests identified the virus as being "not detected" on evaluation. Another subset of the unknown notes were COVID-19 lab requisitions for which no positive/negative indication was provided in the text (i.e. labs were ordered and results were pending or undocumented in the text).

For the health condition diagnosis text stream (Table 4), human review identified 21 documents with a (presumed) positive COVID-19 diagnosis; of which only 12 were identified by COVID-19 biosurveillance algorithm as being COVID-19 positive documents. When writing notes in the health conditions diagnosis text stream, physicians tended to be very brief/terse. For example, the 9 documents which the COVID-19 biosurveillance algorithm did not identify as being positive (but human review did identify as being positive) all took the format: “COVID-19” or "COVID-19 [date]" (which we assumed was indicating the date of a positive COVID-19 diagnosis at the note/patient-level). In these cases, the contextual analyzer and document classification logic did not have enough information to definitively identify whether a note/patient was COVID-19 positive or not; at which point the biosurveillance system defaulted to an “unknown” classification status.

For the clinical notes text stream (Table 4), human review identified 12 COVID-19 positive documents, half of which were identified as being COVID-19 positive by the biosurveillance algorithm. For the clinical notes text stream, the COVID-19 biosurveillance algorithm also identified 241 documents as unknown COVID-19 status. The majority of these documents contained boilerplate text regarding appointments occurring via phone/video/email as a result of the COVID-19 pandemic. Other documents of unknown COVID-19 status contained utterances regarding COVID-19 which pertained to individuals other than the patient (e.g. wife diagnosed with COVID-19) or for which COVID-19 status was uncertain (e.g. individual was symptomatic for COVID-19 and instructed to report to an assessment center for further evaluation).

Overall, the COVID-19 biosurveillance algorithm developed by Chapman et al [15] performed well in identifying COVID-19 positive documents in our primary care setting, making few false positive or false negative errors. Our internal validation study resulted in too few instances of human-labelled/algorithm-predicted COVID-19 positive documents, under a random sampling design, to generate precise estimates of certain algorithm operating characteristics (particularly sensitivity and positive predictive value). In Table 5, we aggregate each of the N=2000 independent documents, across each of the three text streams (1. lab text, 2. health condition diagnosis text, and 3. clinical notes). We assumed "COVID-19 positive" documents were positive; whereas, "COVID-19 negative" documents and "COVID-19 unknown" documents were combined into a single "non-positive/negative" category. Under this assumption, we compute overall operating characteristics of the COVID-19 biosurveillance algorithm developed by Chapman et al [15] in our local study setting. Sensitivity was estimated as 20/35 = 57.1% (95% exact binomial confidence interval: 39.4% - 73.7%); specificity was estimated as 5965/5965 = 100.0% (95% exact binomial confidence interval: 99.9% -100.0%); positive predictive value was estimated as 20/20 = 100.0% (95% exact binomial confidence interval: 83.2% - 100.0%); and negative predictive value was estimated as 5965/5980 = 99.7% (95% exact binomial confidence interval: 99.6% - 99.9%).

### A COVID-19 primary care NLP-derived time series analysis and external validation

To investigate the temporal dynamics of COVID-19 in primary care electronic medical records over the pandemic year we discretized time into 53 weekly bins and summed the number of COVID-19 positive indications/utterances in each week (from either the lab text, health condition diagnosis text or clinical notes). We visualized the resulting primary care NLP-derived series using several time series plots. We further compared the primary care NLP-derived series against other externally generated COVID-19 time series collected/curated by Toronto Public Health, in particular: 1) lab confirmed COVID-19 cases, 2) COVID-19 hospitalizations, 3) COVID-19 ICU admissions, and 4) COVID-19 intubations. The UTOPIAN primary care derived time series of COVID-19 positive utterances illustrated a trend/pattern which appeared strongly correlated with each of the Toronto Public Health COVID-19 time series. In many cases, the series tracked each other strongly, with one series leading/lagging the other during different time periods of the 2020 pandemic year.

## Discussion

In this study we applied a rule-based NLP system to identify COVID-19 positive utterances over millions of clinical text documents, gathered from hundreds of thousands of primary care patient records. The study leverages routinely collected, high quality, primary care clinical notes and demonstrates a novel mechanism for monitoring COVID-19 pandemic impacts on community health. The NLP system was originally developed by the United States Veteran’s Affairs heath system, and we transported the technology without modification to primary care electronic medical records from Toronto, Canada. The rule-based NLP system successfully identified hundreds of distinct COVID-19 entities across three diverse clinical text streams: lab text, health condition diagnosis text and clinical notes. Thousands of primary care patients were identified using the NLP system as having at least one positive COVID-19 document classification over our study timeframe.

The study findings corroborate the important role that primary care physicians have played during the COVID-19 pandemic. Contained within the primary care electronic medical record, there exist thousands of instances where COVID-19 related entities are mentioned in the context of medical communication, disease prevention, disease management and care-planning. A key finding relates to the diversity of COVID-19 entities discovered and the heterogeneity in which these entities are recorded in modern electronic medical record systems. Study findings suggest that COVID-19 utterances are being recorded in numerous areas of the electronic medical record system where clinical narrative data are captured, including: lab text, health condition diagnosis text and clinical notes. Further, there does not appear to be strong agreement between patient-level COVID-19 positivity status across these data sources. This finding suggests that COVID-19 related information is not captured/recorded in a single electronic medical record field, and that studies employing primary care electronic medical records to identify COVID-19 positivity status would be wise to mine all available data sources/streams available, as reliance on a single source is likely to miss important COVID-19 utterances mentioned elsewhere in the patient medical record.

We conducted a manual review and human-labelled thousands of primary care clinical text documents as COVID-19 positive versus COVID-19 negative, permitting estimation of the operating characteristics of the COVID-19 biosurveillance system in our study setting. Our internal validation study demonstrated that the COVID-19 biosurveillance system developed and evaluated by Chapman et al [15] transported well to our setting, maintaining high levels of sensitivity, specificity, positive predictive value and negative predictive value. Our estimate of sensitivity was slightly lower than that of Chapman et al [15]; largely driven by short/terse COVID-19 utterances in the health conditions diagnosis text stream; resulting in uncertain document classification logic and an increase in false negative findings in our study context. Future work will attempt to modify document classification logic for nuances of particular primary care text streams, and extend concept tagging rules to reflect evolving language encountered during the COVID-19 pandemic (e.g. incorporation of novel COVID-19 variants of concern, COVID-19 vaccines, and COVID-19 treatments).

A primary objective of this study was to investigate the temporal dynamics of inferred COVID-19 positivity status from primary care electronic medical records. The estimated COVID-19 primary care NLP-derived time series exhibit temporal dynamics which have strong face validity over the study period. Early in 2020 (prior to the WHO declaring COVID-19 a global pandemic) COVID-19 lab confirmed infections and hospitalizations were low (near zero) in Toronto–this is corroborated in our analyses. In March/April 2020 we observed a sharp increase in COVID-19 positive documents identified in the primary care electronic medical record–the timing being consistent with initial increases in COVID-19 infection in Toronto, Canada. COVID-19 positive document counts displayed a low/minima in the summer of 2020. We note that the largest gap between the COVID-19 NLP derived time series, and the external Toronto Public Health COVID-19 indicator series occurred in the summer of 2020. During this period the COVID-19 NLP series (proportionally) exceeded Toronto Public Health COVID-19 indicator series. We hypothesize this demonstrates how the COVID-19 virus was still impacting local communities (in spite of low public health infection/hospitalization counts), however, was being managed in primary care settings as opposed to being documented in local public health testing centers or hospital settings. Less urgent infections were likely advised to self-isolate (and perhaps did not report their symptoms or health status to public health officials); whereas, only moderate/severe cases presented for testing or acute/emergent care. As lockdown measures were relaxed and public adherence to non-pharmaceutical interventions waned through the fall of 2020, we observed a sharp increase in the number of COVID-19 positive documents/patients recorded in the latter part of 2020. We plotted our COVID-19 derived NLP-series against other important COVID-19 indicator series independently generated by Toronto Public Health using public-health/hospital data sources, including: 1) lab confirmed COVID-19 cases, 2) COVID-19 hospitalizations, 3) COVID-19 ICU admissions, and 4) COVID-19 intubations. On external validation, our NLP-derived COVID-19 primary care series correlated strongly with other important public health series. Strong empirical correlations are encouraging, and future work should continue to investigate whether primary care electronic medical record data can be used to uncover leading indicators of COVID-19 infection/hospitalization–as this type of complementary indicator would greatly assist public health officials and hospital executives attempting to plan for future waves of COVID-19 infection.

A unique aspect of our study is the use of primary care electronic medical record data to investigate COVID-19 positivity status, and temporal dynamics of COVID-19 over the pandemic year. Few other studies/jurisdictions have utilized primary care electronic medical record data for COVID-19 phenotyping or for the identification of leading indicators of COVID-19 infection/hospitalization. The work by Chapman et al [15] illustrates (and provides a working tool) for performing COVID-19 biosurveillance using hospital electronic medical record data. Liu et al [16] developed a COVID-19 hot-spotting algorithm based on the presence/absence of 10 clinical indicators, in the context of a large integrated health system in California, USA. The authors evaluated their algorithm using similar design/methods as our study and found their method to be a strong leading indicator of COVID-19 cases/hospital-admissions. The ability to reconstruct their bespoke algorithm in our primary care setting would be challenging and would require a combination of remapping clinical disease codes to our nomenclature and the use of NLP named entity recognition tools to extract clinical indicators not collected using our standardized nomenclatures. Many other studies have investigated COVID-19 phenotyping using electronic medical record data. Certain exceptional studies include Brat [19] and Klann [20]; however, these studies were developed in the context of hospital electronic medical records, and the availability of requisite data sources for phenotype construction in primary care settings may be limited.

### Limitations

In terms of generalizability, our study included primary care patients from Toronto, Canada. Data were collected from 44 primary care clinics. The sample obtained was slightly older and more likely female, as compared to Toronto population estimates. We did not have access to additional socio-demographic characteristics to characterize the representativeness of our sample. Nonetheless, the COVID-19 biosurveillance methodology feasibly transported to our primary care setting, and inferences regarding the evolution of COVID-19 during the study timeframe validated well when compared to other public health indicator series. Future work will explore expanding this biosurveillance methodology across Ontario/Canada in order to better understand the generalizability of the methodology.

Our internal validation study employed a random sampling design, and resulted in a small number of positive COVID-19 documents. Internal estimates of algorithm operating characteristics (particularly sensitivity and positive predictive value) were imprecise as a result of the low prevalence of COVID-19 over our study timeframe. A larger sample size (resulting in increased resources/costs) would be needed to achieve precise estimates of sensitivity or positive predictive value. However, precise estimates of specificity and negative predictive value were obtained from of our internal validation study.

**Fig 1:**
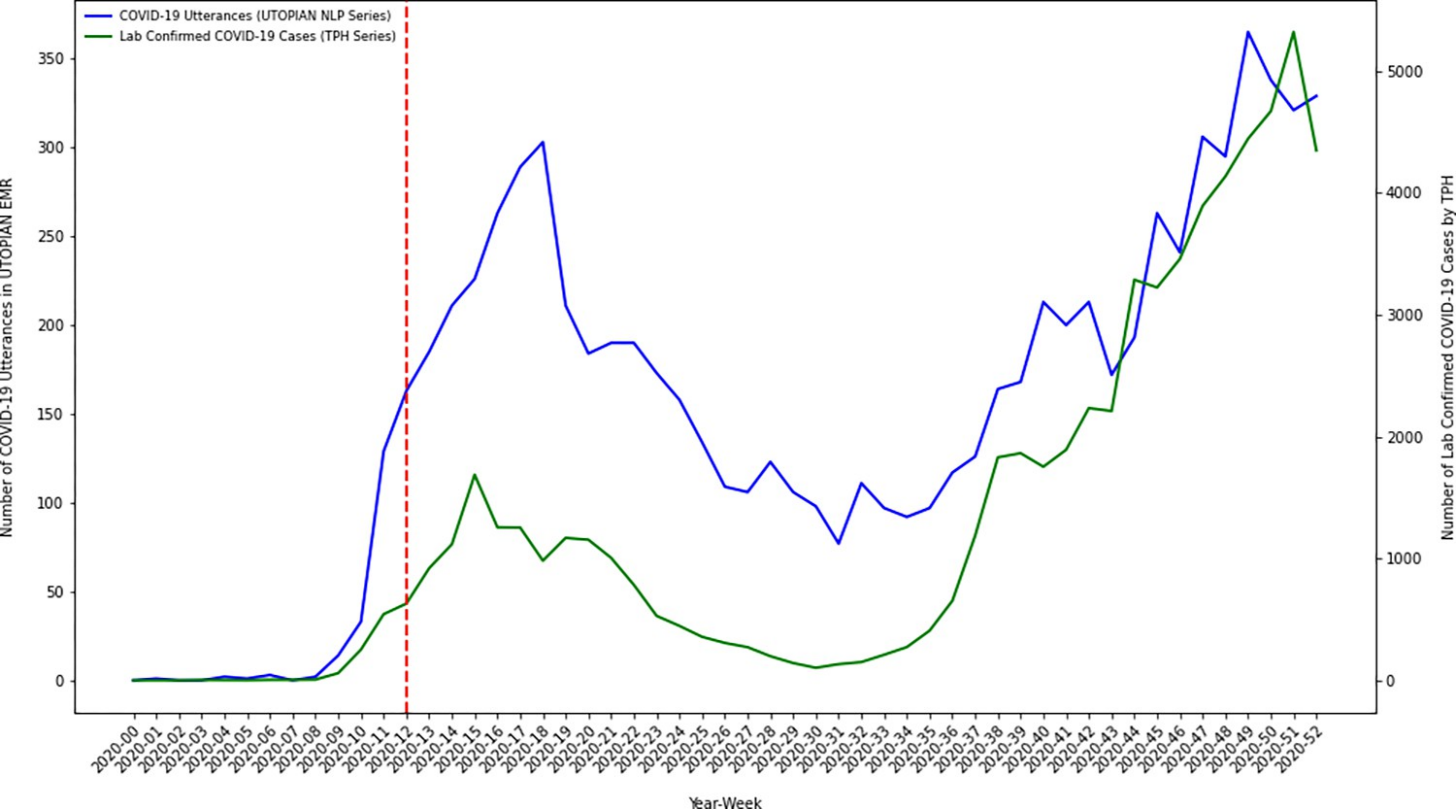
Time series plot of the composite count of the number of COVID-19 positive utterances from each of the three electronic medical record text streams (lab text, health condition diagnosis text and clinical notes) versus the number of lab confirmed COVID-19 cases in Toronto, Canada (data extracted from Toronto Public Health).

**Fig 2:**
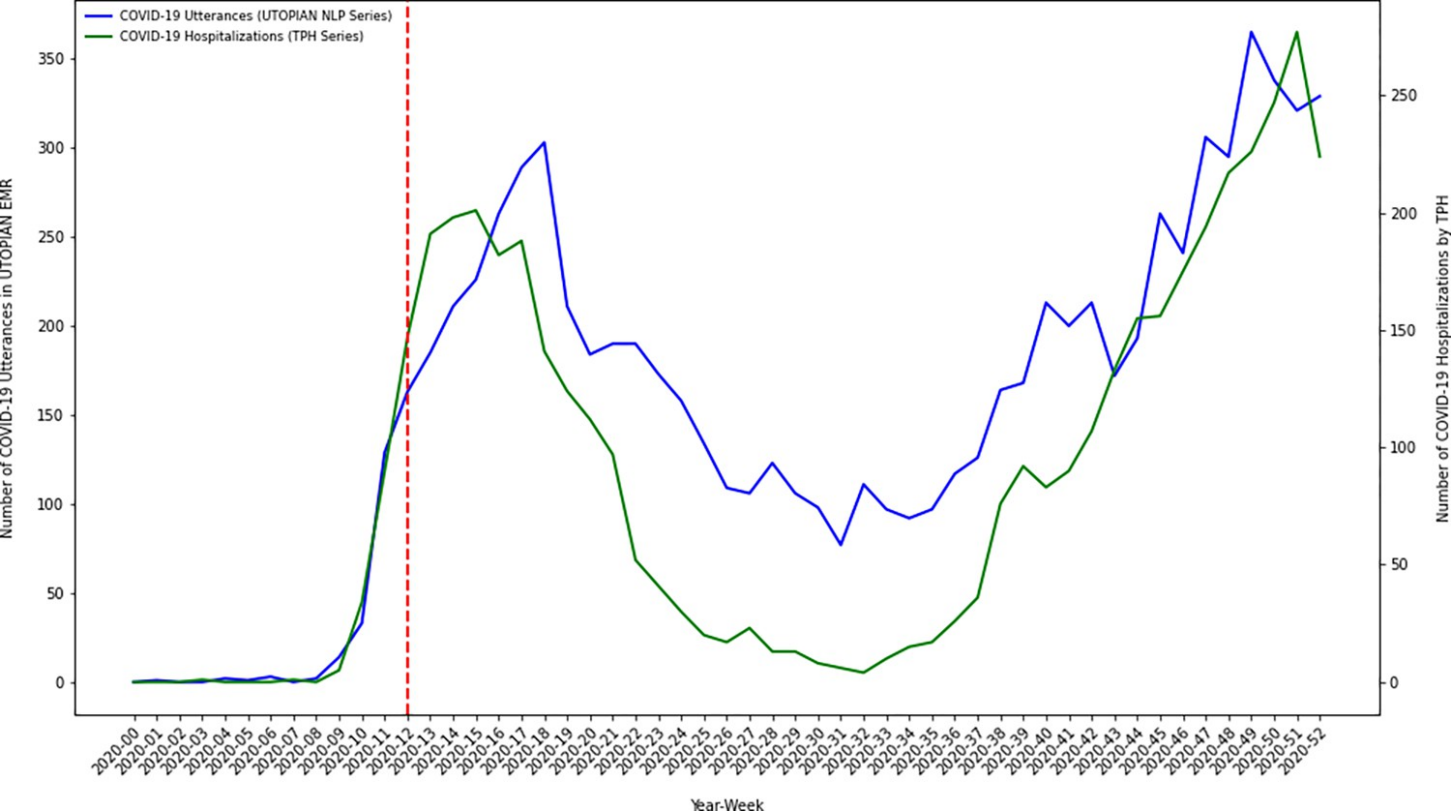
Time series plot of the composite count of the number of COVID-19 positive utterances from each of the three electronic medical record text streams (lab text, health condition diagnosis text and clinical notes) versus the number of COVID-19 related hospital admissions in Toronto, Canada (data extracted from Toronto Public Health).

**Fig 3:**
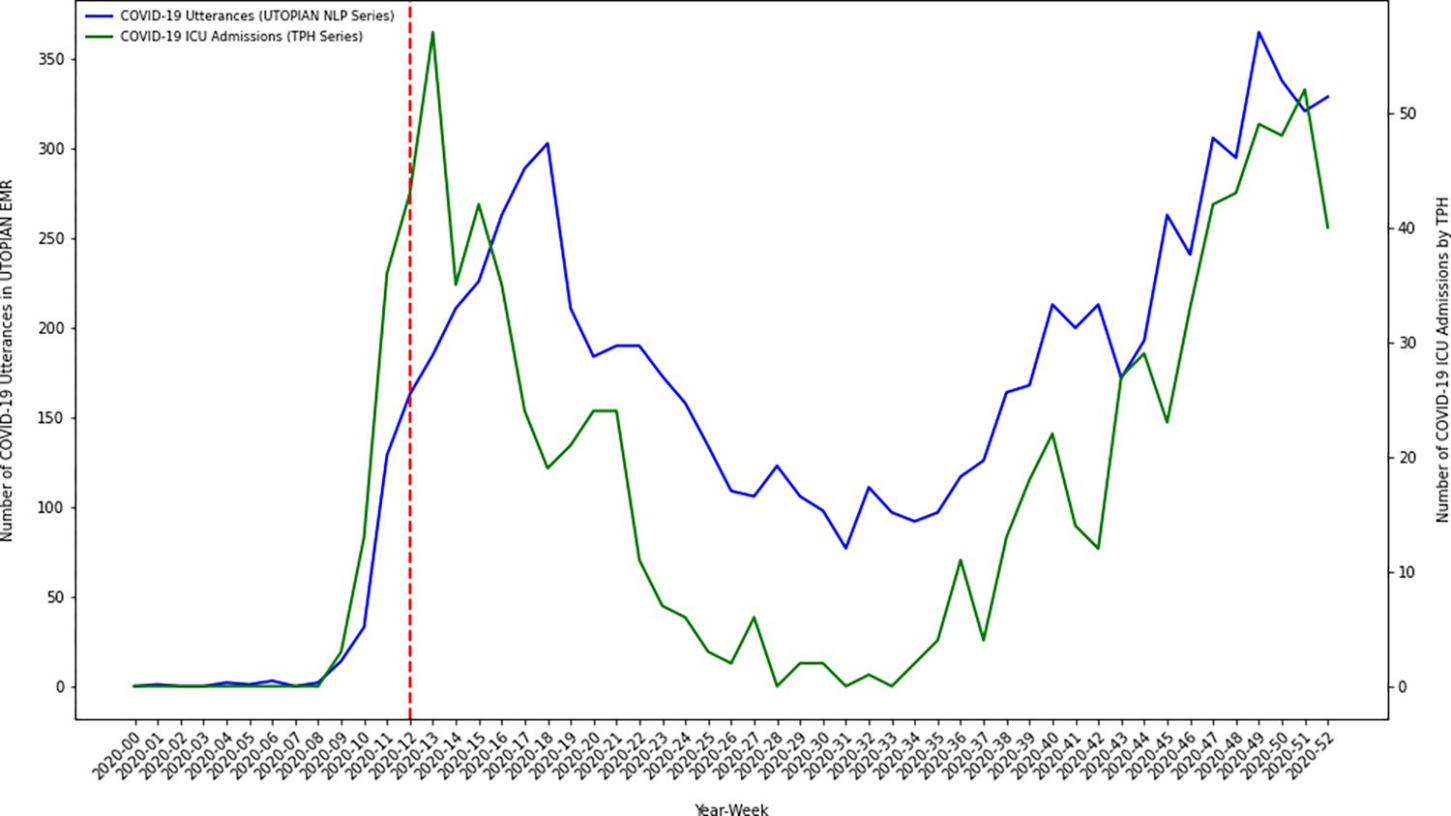
Time series plot of the composite count of the number of COVID-19 positive utterances from each of the three electronic medical record text streams (lab text, health condition diagnosis text and clinical notes) versus the number of COVID-19 related ICU admissions in Toronto, Canada (data extracted from Toronto Public Health).

**Fig 4:**
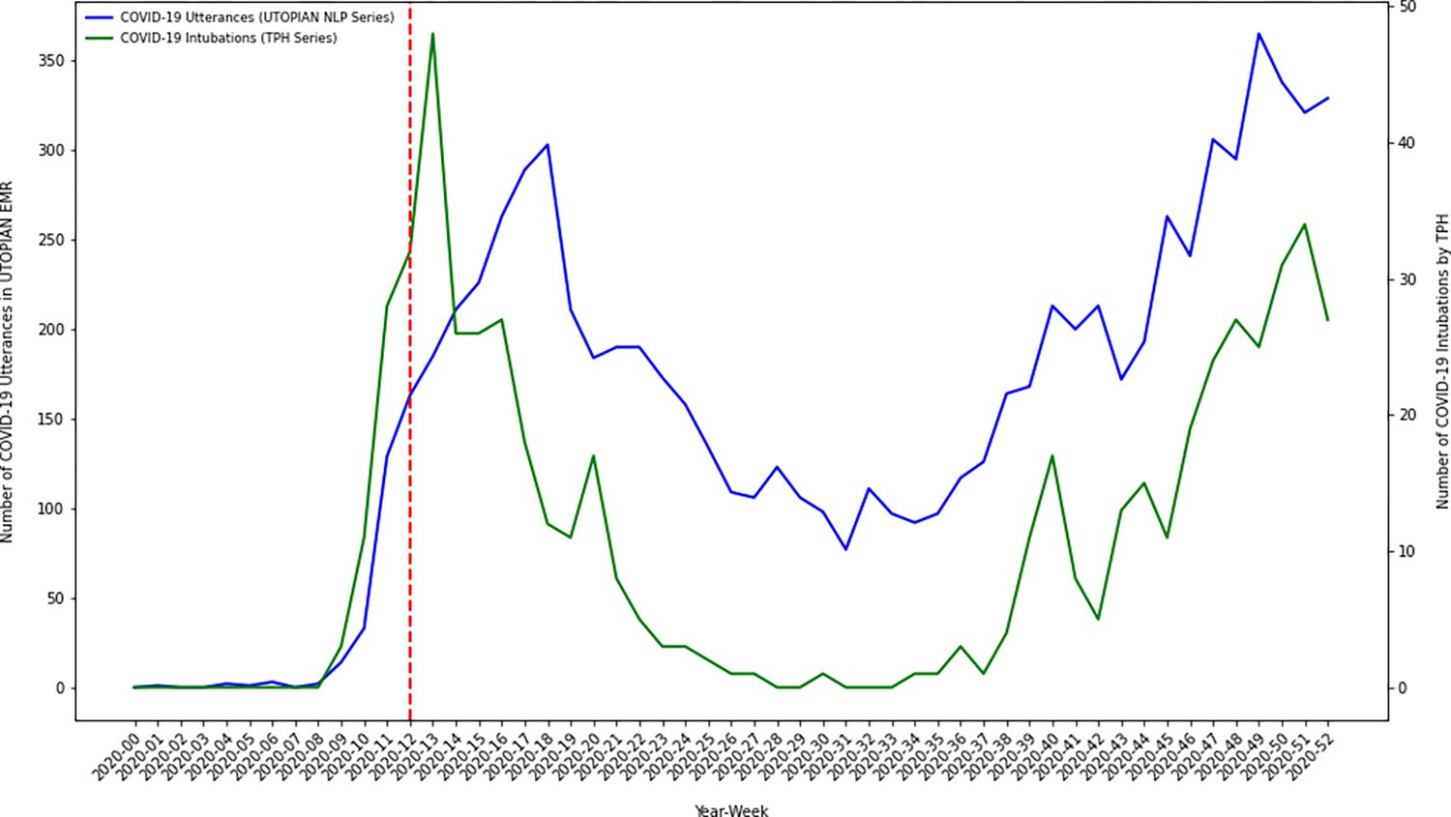
Time series plot of the composite count of the number of COVID-19 positive utterances from each of the three electronic medical record text streams (lab text, health condition diagnosis text and clinical notes) versus the number of COVID-19 related intubations in Toronto, Canada (data extracted from Toronto Public Health).

Inferences regarding the correlation between our primary care text-based indicator series and the Toronto Public Health indicator series were descriptive. On visual inspection of the overlaid series (Figs 1–4) we observed that overall patterns/trends between pairs of series were concordant. Spearman correlation coefficients between the primary care text-based indicator series and the Toronto Public Health indicator series were strong (results not shown). However, when we conducted cross-correlation function analyses between the ARMA(1,1) differenced residual series we did not observe the primary care text-based indicator series as being a consistent leading/lagging indicator of any of the Toronto Public Health indicator series (results not shown). Future work will continue to examine whether for particular COVID-19 waves (subject to particular public health interventions) the primary care text-based series was a leading/lagging indicator of particular public health outcomes.

Our study employed a retrospective design and there existed delay-lags in data reception, curation and reporting. The utility of our findings would be enhanced if more timely access to primary care electronic medical record data existed in our jurisdiction. However, the current primary care research landscape in Canada does not afford for timely access to data (e.g. daily/weekly extractions). Hence even if leading indicators of COVID-19 infection/hospitalization could be identified in primary care data sources, it is not clear whether corporate vendors would participate in a partnership with scientists/researchers and public health organizations that would provide timely access, in a cost-respectful manner, to essential data for COVID-19 monitoring/surveillance.

### Conclusions

In this study, we applied and internally/externally evaluated a rule-based NLP system for COVID-19 biosurveillance using primary care electronic medical records from Toronto, Canada. The method scaled to millions of primary care text documents, and hundreds of thousands of patient records. We identified hundreds of unique COVID-19 entities, and thousands of COVID-19 positive clinical documents, across three primary care clinical text streams. The resulting primary care COVID-19 NLP-derived time series correlated strongly against other important COVID-19 indicator series, externally generated from hospital/public-health data sources. Future work should continue to investigate whether complementary leading indicators of COVID-19 infections or hospitalizations may be uncovered using readily available, passively collected, high quality primary care electronic medical record data.
